# Neuroinflammation, Microglia and Implications for Anti-Inflammatory Treatment in Alzheimer's Disease

**DOI:** 10.4061/2010/732806

**Published:** 2010-06-14

**Authors:** Daniela L. Krause, Norbert Müller

**Affiliations:** Department of Psychiatry and Psychotherapy, Ludwig-Maximilians University Munich, Nußbaumstr. 7, 80336 Munich, Germany

## Abstract

Neuroinflammation has been implicated in the pathology of Alzheimer's disease (AD) for decades. Still it has not been fully understood when and how inflammation arises in the course of AD. Whether inflammation is an underling cause or a resulting condition in AD remains unresolved. Mounting evidence indicates that microglial activation contributes to neuronal damage in neurodegenerative diseases. However, also beneficial aspects of microglial activation have been identified. The purpose of this review is to highlight new insights into the detrimental and beneficial role of neuroinflammation in AD. It is our intention to focus on newer controversies in the field of microglia activation. Precisely, we want to shed light on whether neuroinflammation is associated to brain tissue damage and functional impairment or is there also a damage limiting activity. In regard to this, we discuss the limitations and the advantages of anti-inflammatory treatment options and identify what future implications might result from this underling neuroinflammation for AD therapy.

## 1. Introduction

The pathology of Alzheimer's disease (AD) is characterized by the deposition of amyloid-*β* (A*β*) plaques in the brain parenchyma and neurofibrillary tangles within neurons [1]. 

Apart from the disease's distinct pathological markers, its neurodegenerative conditions are characterized by chronic neuroinflammatory processes. Yet, those inflammatory markers are not exclusively associated with AD. Also brains of “healthy aged” individuals show concentrations of serum markers related to inflammation, homocysteine and cholesterol homeostasis are associated with cognitive functioning in the nondemented healthy aging population [2]. In the AD pathology, these aging-related inflammatory processes are increased. The suggestion that inflammation may participate in AD first came up more than two decades ago. As several clinical trials have shown a beneficial effect for nonsteroidal anti-inflammatory drugs for the occurrence and course of AD, the inflammatory hypothesis in AD gained a lot of attention. In regard to treatment and prevention of AD, several classes of medications have emerged to the market, which improve the cognitive symptoms of this disorder (e.g. the cholinesterase inhibitors). But the relief that these drugs provide remains symptomatic—so it is a major goal for the future to develop effective disease-modifying therapy. Different substantial efforts have been made to identify potential strategies to ameliorate or prevent AD pathology, with data stemming from basic research as well as from animal and epidemiological studies. Because many investigators have concluded that neuroinflammation contributes to neuronal damage in the brain during AD [3, 4], the use of anti-inflammatory drugs as a possible treatment option has been widely investigated [5–7]. Anti-inflammatory therapy has therefore been credited as a strategy for reducing the risk or slowing the progression of AD. However, the results of these studies remain inconsistent [8]. 

Until now, many questions regarding the inflammatory response are still unresolved. Discussion continues whether neuroinflammation is an underling cause or a resulting condition in AD. 

There are several studies showing that an intact immune response including intact T cell immunity is a prerequisite for cognitive function. T cell deficient mice show impaired learning abilities, which can be reversed with T cell substitution [9, 10]. 

Inflammation in the brain is characterized by activation of glial cells (mainly microglia and astrocytes) and expression of key inflammatory mediators as well as neurotoxic free radicals. It has been suggested that neuroinflammation is associated with neurodegenerative disorders—both acute (e.g. stroke, injury) and chronic (e.g. multiple sclerosis, AD). In this context, microglia cells play a crucial role and therefore microglia and cytokines have been extensively studied in these conditions. In the central nervous system, microglia are the resident phagocytes of the innate immune system. Microglia are found in a highly activated state in close anatomical proximity to senile plaques within the AD brain. In this activated state, microglia produce various proinflammatory cytokines and other immune mediators that create a neurotoxic milieu leading to disease progression [4, 11].

The purpose of this review is to highlight our new insights into the role of neuroinflammation in the pathophysiology of AD. It is our intention to focus on newer controversies in the field of microglia activation and its function in AD pathology. For this, we asked ourselves some questions: are neuroinflammatory alterations neuroprotective—or are they rather an underlying cause of AD? And what strategies result from this underling neuroinflammation for future treatment options?

## 2. Characteristics of Neuroinflammation in AD

The relevance of neuroin*ﬂ*ammation to AD pathology has been established by multiple lines of direct and indirect evidence. One argument is that increased microglial activation has been shown in regions associated with A*β* deposition [12]. Upregulated in*ﬂ*ammatory mechanisms colocalize in the AD brain with those regions that exhibit high levels of AD pathology (e.g. frontal and limbic cortex) and are minimal in brain regions with low AD pathologic susceptibility (e.g. cerebellum) [13]. 

As a second point, many of the in*ﬂ*ammatory mechanisms that have been uncovered in the AD brain are established to be cytotoxic in the periphery. Therefore it seems likely that they are also cytotoxic in the brain, an organ that is sensitive to in*ﬂ*ammation (e.g. meningitis, edema). However, inflammation in the brain is different from inflammation in the periphery. AD brains lack the classical hallmarks of inflammation such as neutrophil infiltration and perivascular mononuclear cuffing. As for other neurodegenerative diseases, a local inflammatory reaction is sustained by activated microglia and reactive astrocytes. This is indicated by the presence of antigens associated with microglia activation and inflammatory mediators, such as factors of the complement system, cytokines, and free radicals [14].

For AD a huge variety of proinflammatory markers have been identified, whereas this was not the case for other forms of dementia. A relevant reduction of monocyte chemotactic protein-1 levels in the grey matter in dementia patients has been shown. For interleukin-6 (IL) and related markers of this proinflammatory cytokine system, decreases were observed in the demented population [15, 16]. It is discussed, however, whether this decrease is related to further psychopathological symptoms such as depression [16]. On the other hand, IL-6 has also neuroprotective properties and decreased IL-6 might be associated with decreased neuroprotection [17]. 

Only modest elevations of in*ﬂ*ammatory markers are found in the autopsy of patients lacking a clinical presentation of dementia but who exhibit sufficient A*β* and neurofbrillary tangles to otherwise qualify for the diagnosis of AD. Their level of inflammatory markers is signifcantly greater than levels of nondemented patients, but dramatically less than AD patients [18]. These findings further strengthen that an inflammation is a necessity for clinical symptoms of AD. 

There also is direct evidence of in*ﬂ*ammatory toxicity in the AD brain. For instance, complement fxation and lysis of neurites could be demonstrated ultrastructurally in Alzheimer's disease cortex, but in contrast it was only very weakly detected in nondemented elderly cortex under the same conditions [19]. 

Finally, many clinical and animal studies have strongly suggested that especially nonsteroidal anti-inflammatory drugs (NSAIDs) could be used as preventive or treatment strategies in AD. This aspect is further discussed in a later section of this paper, where we focus on anti-inflammatory treatment.

Even though there are many indicators that neuroinflammation plays a key role in AD pathology, this does not answer which of these inflammatory activities are causing disease progression. The question remains: do some of these processes help to fight against the disease? In order to address this, the role of microglia seems important, because these cells are known for neuroprotective and degenerative functions.

## 3. Controversy: Do Activated Microglia Cause Neuroprotection or -Degeneration in the AD Brain?

Microglia are one of three glial cell types in the central nervous system (CNS). They play an important role as resident immunocompetent and phagocytic cells in the event of injury and disease. Del Rio Hortega determined in 1927 that microglia belong to a distinct glial cell type apart from astrocytes and oligodendrocytes [20]. Since the 1970s there has been wide recognition that microglia are immune effectors in the CNS that respond to pathological conditions and participate in initiation and progression of neurological disorders (including AD) by releasing potentially cytotoxic molecules such as proinflammatory cytokines, reactive oxygen intermediates, proteinases, and complement proteins [21]. This means that their phagocytic function can be beneficial while their inflammation related functions might be detrimental. 

Several studies give evidence for an increased number of morphologically reactive microglia in AD brains compared to nondemented individuals [22, 23]. The location of these reactive microglia has been indentified directly around plaques [24]. This finding has been verified in a recent imaging study, which showed increased microglial activation in regions associated with amyloid deposition [12]. Up to now, the exact timing of this association could not be identified. Microgliosis might be an early component of the disease process and not necessarily dependent upon A*β* plaque interaction as a stimulus. What is known so far is that activation of microglia by A*β* fibrils is associated with a chemotactic response and extensive clustering of microglia around A*β* plaques in the AD brain [25]. These findings indicate the prominent role of microglial cells in AD. Nonetheless it remains unclear, whether their functions are beneficial or detrimental. 

The following section explains the checkered role of activated microglia in AD pathology.

## 4. Neuroprotective Properties of Microglia in AD

Is there a possibility that activated microglia cells are beneficial in neurodegenerative diseases? It is known that the microglia population can be neuroprotective by degrading A*β* plaques in AD. Mouse models found that microglia mainly recruit macrophages from the periphery that then transform into microglia in the brain. Therefore most of the microglia that were associated with plaques in the mouse brain came from the bone marrow [26]. Furthermore it has been suggested that newly recruited microglia have different phagocytotic properties than intrinsic microglia, which is important for A*β* elimination. Lysosmes from the macrophage cell line are more acidic than those of microglial lysosomes [27]. This indicates that microglia derived from the periphery might be more efficient in eliminating A*β* than brain microglia. Furthermore, phagocytic activity of microglia is dampened by proinflammatory cytokines like tumor necrosis factor *α* (TNF) [28]. These findings show that microglia that are committed to an inflammatory response may have a lower phagocytotic capacity, than newly recruited microglia. In mouse models of AD it could be demonstrated that anti-inflammatory drugs like minocycline improve cognitive functions and reduce the activation of microglial cells but do not alter the A*β* plaques deposition and distribution [29]. Seabrook et al. showed in amyloid precursor protein transgenic mice an age dependent effect of minocycline: in young animals the drug increased the amyloid load indicating a beneficial effect of microglia in clearing amyloid [30]. Not only for AD minocycline was investigated as a potential treatment, also in schizophrenia an add-on therapy with minocycline appeared to be effective on the cognitive performance by reducing a broad range of psychotic symptoms [31]. On the other hand an additional mechanism might help microglia cells with the elimination process. Transforming growth factor-*β* 1 has been demonstrated to promote microglial A*β* clearance and reduce plaque burden [32]. This could support the idea that microglial activation is useful in the clearance of A*β*.

A further suggestion for the beneficial role of microglia is that neuroprotection results from the microglial glutamate removal. Glutamate has been indentified as a relevant neurotoxic substance that acts through N-methyl-D-aspartic acid (NMDA) receptors on neurons and can lead to increased neuronal cell death. Microglial cells can increase their capacity to take up glutamate upon stimulation with lipopolysaccaride (LPS) over a mechanism that is TNF*α* dependent [33]. For AD this microglial function could be relevant because memantine (the NMDA receptor antagonist) has been shown to improve cognition, function (activities of daily living), agitation, and delusions in AD patients [34]. Taken this together, microglial cells are important for the control of glutamate levels and might therefore contribute to neuronal survival. There is also evidence that microglia are capable of secreting neurotrophic or neuron survival factors (e.g. nerve growth factor and neurotrophin 3) upon activation via inflammation or injury [35]. 

A recent review explains that microglia—when they are challenged—may adapt to different stimulatory contexts and pass through a sequence of reactive profiles. This is in line with the finding that microglia are not just “resting” but have active sensor and versatile functions [36].

Are most microglial cells functions beneficial in AD? Several studies suggest an overbalance of the detrimental microglial properties. This issue is discussed in the next section.

## 5. Microglia—Are They Responsible for Neurodestruction and -Degeneration?

In order to address this question, it is important to focus on timing. One must investigate when microglial activity begins during the time course of the disease. An increase in microglial activation has been observed in very early stages of AD. This increase surprisingly disappeared over time [37]. The suggestion of Vehmas et al. strengthens the assumption that microglial activation begins early in disease progression [37]. This could be a hint that microglia initially try to eliminate A*β*, but over time of the disease fail and therefore decrease their activity. Alternatively, the microglial role in AD could be detrimental and they initiate the underlying AD pathology. In order to further evaluate this issue, a closer look needs to be taken on what causes the microglial activation in AD and it seems important to distinguish between acute and chronic stimulation of microglial cells. While an acute insult may trigger oxidative and nitrosative stress, it is typically short-lived and unlikely to be harmful to long-term neuronal survival. Therefore it is believed that an acute neuroinflammatory response is generally beneficial to the CNS, since it tends to minimize further injury and contributes to repair of damaged tissue. The opposite is the case for a chronic stimulation: chronic neuroinflammation is most often detrimental and damaging to nervous tissue. Thus, whether neuroinflammation has beneficial or harmful outcomes in the brain may depend critically on the duration of the inflammatory response. The progressive deposition of A*β* in AD disease might provide a chronic stimulus to microglial cells. Also the chemotactic functions of A*β* to attract microglia contribute further to the ongoing inflammatory process [25]. The ratio of the proinflammatory cytokine IL-1*β* to the anti-inflammatory cytokine IL-10 is drastically elevated in the serum of AD patients, giving these patients a definite long-term proinflammatory profile [38], indicating a chronic neuroinflammatory state of the CNS. In addition, the accumulating loss of neurons that characterizes AD further contributes to generation of debris and keeps microglia activated indefinitely maintaining microglia in an activated state long term. This data indicates that in AD the inflammation might be rather chronic and therefore contributing to disease progression. 

There is also the emerging idea that an inflamed CNS environment may influence the ability of microglia to contribute to plaque deposition rather than plaque removal [28]. This strongly suggests that the microenvironment of the brain can influence whether microglia perform beneficial or deleterious functions in pathophysiological states. This means that microglia cells functionally adapt to their environment [36]. Recent studies show that in response to certain environmental toxins and endogenous proteins, microglia can enter an overactivated state and release reactive oxygen species (ROS) that cause neurotoxicity [39]. Overactivated microglia can be detected using imaging techniques and therefore this knowledge offers an opportunity not only for early diagnosis, but eventually also for the development of targeted anti-inflammatory therapies that might diminish the progression of the disease [21].

In addition, activated microglia release the excitotoxin quinolinic acid [40], and microglia activated by AD plaques produce an apparently novel amine that evokes fulminant excitotoxicity [41]. One interesting implication of an excitotoxic contribution to in*ﬂ*ammatory mechanisms is the potential for limited damage to functional cellular compartments. Because excitatory amino acid receptors are restricted to synapses and dendrites, these subcellular compartments are preferentially vulnerable. 

As a result, microglia-produced excitotoxins may lead to cognitive impairment that is not necessarily correlated with neuronal cell loss [3]. However, activated microglia do not only produce neurotoxic metabolites. Some of their products like 3-hydroxyanthralinic acid (which is—like quinolinic acid—one of the downstream products of the tryptophan metabolism) exert antioxidant and anti-inflammatory functions [42, 43]. Therefore the balance of these products that result from activated microglia is important for the inflammation process. 

**Figure 1:**
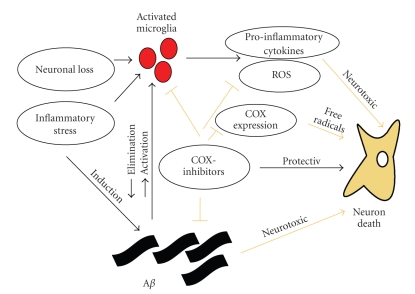
possible interactions of COX-inhibitors and Alzheimer‘s disease pathology. The fair arrows show neurotoxic properties of A*β*, COX-expression cytokines. In addition it is indicated that COX-inhibitors block COX-expression, activated microglia, ROS, and A*β*. ROS: radical oxygen species; COX: cyclooxygenase; A*β*: amyloid *β*.

To sum up the results from microglial studies: clear indications for the important role of neuroinflammation contributing to disease progression in AD were found. However, some parts of microglial activation might also be beneficial during the course of AD. These issues are shown in Figure 1.

## 6. The Role of COX Inhibitors in Neurodegeneration

As explained above, neuroinflammation is a critical event in AD. It has been suggested that anti-in*ﬂ*ammatory therapy could be benefcial in delaying the onset or slowing the progression of AD.

Cyclooxygenase (COX) is a unique enzyme. First, it exhibits two catalytic activities, a bis-oxygenase activity, which catalyses prostaglandin G_2_ (PG) formation from arachidonic acid and a peroxidase activity, which reduces PG G_2_ to PG H_2_. The peroxidase activity also results in the production of free radicals, which are in part utilized by COX itself [44]. Although NSAIDs may have other effects as well, it is generally assumed that their primary mechanism of action is by competitive inhibition of COX activity, thereby reducing the production of in*ﬂ*ammatory prostaglandins from membrane-derived arachidonate. COX not only helps mediate production of prostaglandins and other in*ﬂ*ammatory factors, it is itself upregulated by pro-in*ﬂ*ammatory mediators [44]. 

In AD, A*β* neurotoxicity may result from several mechanisms, most likely in combination. These mechanisms include oxidative damage, direct cytotoxicity, and induction of destructive inflammatory mechanisms; efforts have been directed at the control of each of these processes [45]. See Figure 1 for the involvment of COX in the AD pathology. 

The treatment of AD with NSAIDs is one of the most promising approaches.

## 7. Possible Mechanisms of Action of NSAID in AD

If NSAIDs are beneficial in AD, the presumed mechanism would be inhibition of COX expressed in the brain. Both COX-1 and COX-2 are expressed there and COX-2 plays a unique role in the brain compared to the periphery: only in the brain COX-2 is expressed constitutively whereas elsewhere the expression is activation-dependent. Although in vivo the majority of COX-2 appears to be made in neurons, COX-2 was also seen in rat astrocytes and microglia [46]. It has been demonstrated that COX-inhibiting NSAIDs reduce microglial activation following infusion of A*β* in rats [47]. Neuronal stress, such as ischaemia and excitotoxicity, is associated with strong upregulation of neuronal COX-2 expression. This suggests that COX-2 is involved in neurotoxic mechanisms and may therefore represent a target for drug therapy in the treatment of AD [48, 49]. 

Several studies provide the background for possible mechanisms of action of NSAIDs in AD. Neuronal COX-2 is upregulated in response to exposure to A*β* [50], and focal increases in COX-2 have been shown in the region of amyloid plaques in double transgenic mice carrying genes that encode both mutant APP and mutant presenilin 1 [51]. Many studies seem to show that COX-2 inhibition confers neuroprotection [52–55]. Some studies have revealed an upregulation of neuronal COX-2 in the brains of patients with AD [56, 57], though this has not been a universal finding [58, 59]. One explanation for the variation of COX expression is the short half-life of COX-2 transcripts or individual variability of inflammatory-related processes.

Another principle of how NSAIDs could act, comes from the finding that prostaglandin E2 levels are elevated in patients with AD, especially in early stages of the disease [60]. Therefore NSAIDs blocking prostaglandin E2 synthesis might be beneficial. This issue is further strengthened by glial culture studies indicating that prostaglandins, particularly prostaglandin E, alter the production of several in*ﬂ*ammation-related molecules, including IL-6, chemokines, and APP [61–63]. 

In addition to the more traditional in*ﬂ*ammatory mechanisms associated with COX, unique functions of COX-mediated damage may also occur in the AD brain. For example, several of the prostanoid products of arachidonate metabolism potentiate glutamate excitotoxicity, and COX-2 overexpressing transgenic mice exhibit increased neuronal susceptibility to excitotoxic insult [64]. 

Some of the previously mentioned studies of COX in ischemia also suggest that intraneuronal COX-2 levels may contribute to neuronal death by production of free radicals [65]. In addition, increased COX-2 levels in AD neurons may directly damage neurons or increase their vulnerability to other detrimental processes occurring in AD brain [65]. Thus, NSAIDs actions to inhibit COX-mediated production of apoptotic factors by neurons could be one of the mechanisms by which these drugs seem to exert benefcial effects in AD. 

Another non-COX-dependent mechanism of NSAIDs is to attenuate in*ﬂ*ammatory processes in a manner by directly activating the peroxisome proliferator-activated receptor gamma (PPAR*γ*), a receptor and nuclear transcription factor [66–68]. PPAR*γ* is a member of the orphan nuclear receptor family and in cells of monocytic lineage, including microglia, acts to suppress the expression of a broad range of proin*ﬂ*ammatory genes [66, 68]. Some NSAIDs act as PPAR*γ* agonists, directly binding to it and initiating its transcriptional activity. Activation of PPAR*γ* inhibits the A*β*-stimulated activation of microglia and monocytes and their secretion of proin*ﬂ*ammatory and neurotoxic products. For example, PPAR*γ* agonists act to inhibit the A*β*-stimulated expression of IL-6 and TNF-alpha [69], by microglia and monocytes, and to prevent A*β*-mediated conversion of microglia into an activated phenotype [70].

A further underlying mechanism of AD pathology is oxidative stress [71, 72]. Activated microglial cells are known to release ROS, which might possibly cause this oxidative stress. Though glia cells can also exhibit antioxidative functions by releasing hemeoxygenase-1 (HO-1) triggered by accumulation of 3-hydroxyanthralinic acid (3-HAA), a down-stream product of the tryptophan metabolism. The association of neuronal injury in AD and oxidative stress has been demonstrated by overexpression of immunoreactive HO-1 protein in neurons and astrocytes of the cerebral cortex and hippocampus. HO-1 was found to be colocalized to senile plaques, neurofibrillary tangles, and corpora amylacea [73]. It is widely accepted that a moderate activation of heme catabolism is neuroprotective and contributes to degradation of neurotoxic protein aggregates. Regulatory interactions between HO-1 and COX pathways have also been reported [74]. However, experimental observations indicate that the extent of HO-1 induction may be critical because excessive heme degradation may result in toxic levels of carbon monoxide, bilirubin and iron. Pharmacological modulation of HO-1 levels in the brain shows promising results in models of AD and Parkinson's disease [75]. 

Referring to the oxidative stress underlying AD pathology, one further aspect of these reactive oxygen species includes activation of COX-1/2, which are blocked by NSAIDs. It has been shown that daily doses of NSAIDs increase circulating levels of antioxidants [76]. In a rat model of AD it was suggested that treatment with a COX-2 inhibitor reduces oxidative stress and might therefore be beneficial for the course of AD [77].

As another mechanism it has been suggested that NSAIDs directly affect amyloid pathology in the brain by reducing A*β*-42 peptide levels over the gamma-secretase activity independently of COX activity [78]. Weggen et al. reported that the NSAIDs ibuprofen, indomethacin, and sulindac sulphide preferentially decrease the highly amyloidogenic A*β*-42 peptide produced from a variety of cultured cells by as much as 80% [79]. However, for some NSAIDs the lowering effect of A*β*-42 could not be shown; instead, an increase in A*β*-42 levels was observed [80]. The underlying mechanism of how NSAIDs decrease A*β*-42 was clarified by Lleo et al., who demonstrated that A*β*-42 lowering NSAIDs specifically affect the proximity between APP and presenilin 1 and alter a novel allosteric mechanism of action [81].

## 8. Anti-Inflammatory Treatment Studies in AD

In recent years it has become widely accepted that inflammatory processes are an underlying condition of AD. Therefore a number of clinical trials investigating different anti-inflammatory treatment regimens have been performed. In the following paragraph, we summarize the most import findings in regard to first mainly COX-2 dominant and second COX-1 inhibitors.

A prospective cohort study with 6989 subjects showed that long-term use of NSAIDs protects against AD but not against vascular dementia [5]. More recently, Szekely et al. provided very similar findings: they concluded that NSAIDs use reduced the risk of preferentially AD versus vascular dementia but mainly in those individuals having an apolipoprotein E (APO) epsilon 4 allele. This study was done with over 3,000 subjects aged 65 years and older [6]. Not only selective COX-2 inhibitors were shown to be associated with decreased risk of AD; a reduced occurrence of AD could also be demonstrated for the use of the COX-1 inhibitor aspirin [7]. A meta-analysis of 17 epidemiological studies yielded strong, generally consistent, statistical evidence that NSAID and steroid use is associated with reduced risk of AD [82]. Vlad et al. investigated 49,349 patients with AD and 196,850 controls: long-term (> 5 years) nonsteroidal anti-inflammatory drug use was shown to be protective against Alzheimer disease. These findings were clearest for ibuprofen, but did not appear for other NSAIDs [83].

Not all studies showed a positive outcome for COX inhibitors in AD patients: the failure of selective COX-2 inhibition (rofecoxib) over placebo was stated in a one-year randomized controlled study. The authors argued that their results could indicate that the disease process was too advanced to be modified, as the goal of the study was slowing the progression of dementia in patients with already established AD [8]. For another COX-2 inhibitor, celecoxib, no beneficial effect on the occurrence of AD could be demonstrated in an age group over 70 years [84]. Also Wolfson et al. looked retrospectively at a case control population and found no support for a beneficial effect for NSAIDs in the AD subjects [85]. However, this negative result may have been caused by an insufficient period of data collection before disease onset.

## 9. Conclusion

It is indisputable that neuroin*ﬂ*ammation plays a key role in AD pathology. Mechanisms that parallel those encountered in localized peripheral in*ﬂ*ammatory responses are readily identifed, along with detailed pathways for how the mechanisms interact. On balance, it is likely that AD neuroin*ﬂ*ammation exacerbates AD pathogenesis. 

A general treatment principle in psychiatry, that an intervention as early as possible leads to the best outcome, seems to be especially true for AD. Many lines of evidence show that A*β*-induced neuroinflammation is an early event in neurodegeneration of AD [86], as increases in microglial activation has been observed in very early stages of AD and disappeared over time [37]. The fact that neuroinflammation occurs very early in AD could explain why anti-inflammatory treatment seems to be most efficient as preventive or early treatment. There are several reasons why an early use of NSAIDs is superior to a late one: Cox-expression in the brain decreases over time in AD brains [87]. And the CSF PG E_2_ levels in patients with Alzheimer's disease were high when their short-term memory scores were just below those of controls, but were low in later stages of the disease. These findings further support that inflammatory processes predominate early in Alzheimer's disease [88] and therefore require early intervention with anti-inflammatory treatment.

This could explain the failure of some prospective clinical trials of selective COX-2 inhibitors: it may be related to a delayed onset of treatment, but eventually also to drug selection (regarding different effects of COX-1 and COX-2) and dose and duration of treatment. Especially the drug selection seems essential as some NSAIDs have recently been shown to increase A*β*-42 levels [77]. It also has to be noted that the protective effects of NSAIDs may be via non-COX-inhibitory mechanisms, such as lowering of A*β* levels and activation of the peroxisome proliferator-activated receptor-[gamma] [89] and these non-COX-dependent mechanisms might be differentially distributed among COX-inhibitors.

However, two major aspects should be kept in mind when considering the significance of COX-2 activity in brain diseases. The first thing: COX-2 is expressed under normal conditions and contributes to fundamental brain functions such as synaptic activity, memory consolidation, and functional hyperemia. The second thing: the term neuroinflammation is a much more controlled reaction than inflammation in peripheral tissues. In degenerative diseases, it mainly occurs in the absence of blood-borne infiltrating cells and is sustained by activated glial cells, particularly microglia. 

In summary, the harmful inflammatory processes seem to dominate AD pathology, but there are also some beneficial functions for inflammatory subsets. If AD neuroinfammation is approached with realistic expectations and rational drug design, AD patients could significantly benefit from anti-in*ﬂ*ammatory treatment, especially with NSAIDs. 

A future goal could be to utilize not only the efficient treatment properties of NSAIDs in early AD, but also makes use of the neuroprotective aspects of neuroinflammation with a combination therapy that maximizes the potential of glial activation. This would include treatment with NSAIDs and drugs that enforce anti-inflammatory and antioxidative properties (e.g. with 3-HAA and HO-1 enhancement).
